# MaxEnt modeling and risk evaluation of chagas disease vectors in the domestic cycle of Hidalgo, Mexico

**DOI:** 10.1371/journal.pntd.0013199

**Published:** 2025-07-31

**Authors:** Mónica Chico-Avelino, Josefina Ramos-Frías, Adriana López-Mejía, Santiago Martínez-Calvillo, Rebeca Georgina Manning-Cela

**Affiliations:** 1 Departamento de Biomedicina Molecular, Centro de Investigación y de Estudios Avanzados del IPN, Mexico City, México; 2 Laboratorio de Sistemas de Información Geográfica y Análisis Espacial. Facultad de Estudios Superiores Iztacala (FESI), Universidad Nacional Autónoma de México (UNAM), Tlalnepantla, México; 3 Coordinación Estatal de Vectores, Secretaría de Salud de Hidalgo, Pachuca, México; 4 Unidad de Biomedicina, Facultad de Estudios Superiores Iztacala (FESI), Universidad Nacional Autónoma de México (UNAM), Tlalnepantla, México; Tulane University School of Public Health and Tropical Medicine, UNITED STATES OF AMERICA

## Abstract

This study developed MaxEnt models to determine the potential distribution of four triatomine vector species of Chagas disease in the domestic cycle in Hidalgo state, Mexico: *Triatoma dimidiata* (Latreille, 1811), *T. mexicana* (Herrich-Schaeffeer, 1848), *T. gerstaeckeri* (Stål, 1859), and *T. barberi* (Usinger, 1939). We analyzed over 500 occurrence records alongside selected bioclimatic, topographic, and land cover variables. Key determinants influencing each species’ distribution included climate types, altitude, and anthropogenic factors. Model validation used statistical methods with Area Under the Curve (AUC) metrics, where AUCs ≥ 0.8 indicated good performance, along with experimental validation performed for the first time in the context of Chagas disease through targeted field collections at predicted sites. The results showed high concordance between model classifications and field data, confirming the models’ validity. The identified suitable habitat areas correlated with known ranges of the vector species, providing insights into Chagas disease transmission risk in the domestic cycle. This integrated approach not only validated the presence and absence of the modeled species but also documented the current presence of three vector species, enhancing our understanding of factors influencing vector distributions. Ultimately, this research aims to inform epidemiological control efforts and improve Chagas disease surveillance strategies.

## Author summary

This study used MaxEnt modeling to develop potential distribution models for the four main vectors of Chagas disease in the domestic cycle of Hidalgo, Mexico: *T. dimidiata*, *T. mexicana*, *T. gerstaeckeri*, and *T. barberi*. We analyzed over 500 occurrence records alongside environmental variables. The models were validated statistically using AUC metrics (AUCs ≥ 0.8) and experimentally through field collections at selected sites. Validation showed high concordance between model predictions and field data. Climate types were the primary contributors to most models, with precipitation and altitude also influencing some species. This integrated approach enhanced our understanding of vector habitats within environmental gradients in Hidalgo, providing valuable insights for Chagas disease surveillance and control efforts.

## Introduction

Chagas disease is a significant public health challenge in Latin America. Caused by the protozoan parasite *Trypanosoma cruzi* and primarily transmitted by triatomine bugs, the disease affects approximately 6–7 million people worldwide and is classified as a neglected tropical disease by the WHO [1]. Triatomines are blood-feeding insects, with 152 species described globally (comprising 16 genera, including two fossils) [2,3]. They are found throughout the Americas, from the southern United States to northern Chile and Argentina [4]. In endemic regions, triatomine species inhabit rural, suburban, and unsanitary urban households, which increases human contact and parasite transmission, often linked to inadequate socioeconomic and cultural factors [5,6]. Different species also exhibit varying adaptability to human-altered environments and geographic dispersion, influencing vector-human interactions [7]. Therefore, understanding vector distribution patterns is crucial for managing transmission risk in areas where Chagas disease persists. Analyzing the distribution patterns of vector species and the environmental factors influencing them provides valuable insights into the spatial conditions that promote or restrict their presence. Consequently, potential distribution models are increasingly relevant for understanding the epidemiology of vector-borne diseases, as they can elucidate these relationships [8–10]. These models utilize algorithms to generate potential distribution scenarios based on known occurrence data and environmental variables. Occurrence data can be sourced from historical records, taxonomic collections, digital databases, and field observations. Common environmental predictors include geological, topographic, and climatic variables, which are assumed to influence distributions either individually or collectively. Some models also incorporate socio-demographic factors [11,12]. From these data, potential distribution models analyze patterns of species presence and absence by relating documented locations to explanatory covariates [13,14]. By linking occurrence records to these factors, the models identify regions that may be suitable but have yet to be surveyed, thereby aiding targeted fieldwork and enhancing our understanding of the key factors shaping a species’ geographic range [13]. Areas of high suitability represent potential habitats where conditions resemble those of documented locations, based on specified predictors [15]. Potential distribution models have been employed to estimate high-probability distribution areas, indicating potential epidemiological risk sites for different vector species. For instance, models have been developed for various mosquito genera, including *Lutzomyia* vectors of leishmaniasis [16] and *Culex pipiens*, a vector of West Nile virus [17]. The *Aedes* genus, which includes vectors of dengue virus [18–21], and the *Anopheles* vector of malaria [22,23] have also been studied. This approach has been applied to Chagas disease to assess the risk of *T. cruzi* transmission by identifying potential areas of insect vector presence [2,7,24–30]. Potential distribution models have analyzed the relationships between triatomine distributions and factors at various spatial scales. At the global scale, models have identified climatically suitable sites for 14 species, showing a greater potential for their presence in tropical and subtropical regions [2]. In Latin America, several modeling studies on Chagas disease vectors have been conducted. For example, *T. maculata* (Erichson, 1848) shows broad environmental suitability from Panama to northern Brazil, while *Rhodnius pallescens* (Barber, 1932) has a more restricted distribution, mainly along the coastal areas of Costa Rica and parts of Nicaragua, Honduras, Belize, the Yucatan Peninsula, and northern Colombia and Brazil [24]. The relationship between ecological niche and physiological thermal limits has been studied in seven triatomine species, revealing that thermal tolerance increases with latitude, primarily due to enhanced cold tolerance. Species in southern regions exhibit greater thermal tolerance than those in tropical areas [25]. Additionally, geostatistical modeling across the Americas has estimated ranges for four Chagas disease vector species, suggesting that *T. gerstaeckeri* may be widely distributed from the US to Mexico, while *T. dimidiata* shows potential distribution throughout Mexico, Central America, and northern Venezuela and Colombia [7]. In Mexico, ecological niche models have been developed for 19 vector species and *T. cruzi*. The species with the widest potential distribution were found in the Nearctic Region, while those in the Nearctic and Neotropical regions exhibited smaller potential areas of presence, especially with less extensive probabilities in the latter. Notably, three of the 19 species (*T. longipennis*, *T. mexicana*, and *T. barberi*) overlapped with the highest number of human communities, indicating a high level of exposure to infection [26]. Finer-scale modeling has mapped the probable presence of *T. recurva* (Stål, 1868) in northern Mexico [27]. Another study developed ecological niche models for both *Dipetalogaster maxima* (Uhler, 1894) and *T. cruzi*, identifying exposure risks from potentially infected bites along western and southern coastal regions of Baja California, including a highly touristic site as a high-risk area [28]. In central Mexico’s Guanajuato state, ecological niche models have predicted the distribution of five vector species, with the highest probability of presence concentrated in the Sierra Gorda region, where *T. mexicana* and *T. barberi* showed the greatest extent of probability [29]. Lastly, a predictive model identified potential Chagas disease transmission risk areas in southeastern Mexico’s Yucatan Peninsula, based on the abundance of *T. dimidiata* and *T. cruzi* infection rates [30]. Model validation statistically assesses the performance and classification ability to discriminate between input data (e.g., presences, absences, pseudoabsences) and independent contrast data. Most researchers prefer using independent data, though the same data used to generate the model can also be an option when independent data is unavailable [31]. As mentioned, potential distribution models are derived from publicly accessible online biological databases or other sources containing records; few studies have specifically sampled to estimate an organism’s distribution [32,33]. Ideally, targeted field searches guided by the probabilities generated from potential distribution models should be employed for experimental validation. This approach has been effective for both botanical and animal species, improving species sampling design by validating the models’ classification abilities [34–36]. However, this methodology is not common and appears to have been underutilized in Chagas disease vector studies. Consequently, validating potential distribution models remains challenging due to limitations in the availability of occurrence data and efforts for direct ground-truthing of distribution representations.

The state of Hidalgo, Mexico, is endemic for Chagas disease, with varying levels of seroprevalence reported in the general population (3.25–8.21%) and among blood donors (0.73–5.9%) [37–42]. Recently, we reported the presence of seven synanthropic triatomine species in Hidalgo, including three observed for the first time in the state: *T. nitida* (Usinger, 1939), *T. pallidipennis* (Stål, 1872), and *T. phyllosoma* (Burmeister, 1835). The predominant species (*T. dimidiata*, *T. mexicana*, *T. gerstaeckeri*, and *T. barberi*) displayed a differential distribution pattern with a statistical association to environmental characteristics such as climate variables, temperature ranges, and precipitation levels across the state [43]. The presence of *T. longipennis* was also recently reported in the state [44,45]. Building on these findings, the current study aims to develop MaxEnt models to better understand the potential distributions of these four vector species. We hypothesize that their distributions are influenced by bioclimatic, topographic, and land use variables, which can be modeled using MaxEnt. Therefore, we seek to determine the potential distribution of *T. dimidiata*, *T. mexicana*, *T. gerstaeckeri*, and *T. barberi* in the domestic transmission cycle of Hidalgo by developing MaxEnt models that incorporate over 22 years of occurrence records and selected geoclimatic variables. Model validation will be conducted statistically by calculating the Area Under the Curve (AUC) generated by MaxEnt version 3.4.1 [46] and NicheToolBox [47] values, as well as experimentally through field validation (*in situ*) of absence and presence sites in the domestic cycle based on potential distributions identified by the models, considering previously documented seasonal triatomine patterns [43]. Together, the modeled potential distributions and the statistical and field validation efforts will provide greater insight into the geographic ranges of these important Chagas disease vectors in the domestic cycle of Hidalgo state.

## Materials and methods

### Triatomine species occurrence database

Occurrence data for *T. dimidiata* (260 unique occurrences), *T. mexicana* (154 unique occurrences), *T. gerstaeckeri* (94 unique occurrences), and *T. barberi* (21 unique occurrences) in Hidalgo state, Mexico, were collected by vector personnel of the health services during the period 1997–2019. The data were obtained from: 1) the Epidemiological Reference Institute (InDRE), accessed through the Global Biodiversity Information Facility (GBIF), which aggregates occurrence records from various datasets (https://www.gbif.org/es/), and 2) records provided by the state Vector Control Program of the Secretary of Health of Hidalgo (VCPH). Both sources used the same man-hour per house sampling effort, following Mexican Standard NOM-032-SSA2–2014 (https://www.dof.gob.mx/nota_detalle.php?codigo=5389045&fecha=16/04/2015#gsc.tab=0). Triatomines found by the community are also included. These datasets were previously processed, curated, georeferenced, validated, and published by our research group [43]. All occurrence records from both sources underwent standardization and quality control procedures before being combined for potential distribution modeling of the four dominant triatomine vector species found within the domestic cycle of Hidalgo. This process ensured high data quality and consistency across the datasets.

### Explanatory variables

Based on a review of the literature regarding potential distribution models for Chagas disease vectors, we compiled maps of 23 geo-climatic and anthropic variables commonly used in these models [2,7,11,27,48–51]. The 23 bioclimatic variables from WorldClim (https://www.worldclim.org/) were resampled from a 1 km resolution to 60 m for Hidalgo state, using high-resolution climate data for Mexico from 1980-2009 [52]. This dataset included monthly precipitation and maximum/minimum temperatures, facilitating eco-epidemiological analysis and risk mapping. Variable values were extracted at regularly spaced points across Hidalgo, generating raster layers for each climatic factor through Inverse Distance Weighted (IDW) interpolation. This geo-statistical technique estimated values for unsampled pixels based on known data from surrounding points, with greater weights assigned to closer distances [53]. Additional variables included climate type maps of Mexico, modified by García at a scale of 1:1,000,000 from the National Commission for the Knowledge and Use of Biodiversity (CONABIO), altitude data from Mexico’s National Elevations Continuum (CEM) at a scale of 1:50,000 from National Institute of Statistics and Geography, and land use/vegetation set VI from 2016 maps at a scale of 1:250,000 from the National Institute of Statistics and Geography (INEGI). Vegetation quality was assessed using the Normalized Difference Vegetation Index (NDVI) derived from Landsat 8 OLI multispectral imagery from 2020 at a scale of 1:100,000, which utilizes the differential reflectance of red and infrared light to indicate photosynthetic activity [54]. All layers were projected to the UTM WGS84 14N coordinate reference system at a resolution of 60 m. Cartographic processing was conducted using QGIS 3.22 software. Together, these resampled bioclimatic, topographic, and land cover variables captured influences on vector habitat suitability at finer scales within Hidalgo state.

### Selection of explanatory variables

To develop optimal potential distribution models with strong classification abilities for identifying potential presence and absence sites, a subset of the 23 environmental variables was selected based on their explanatory power. Initial MaxEnt runs generated two submodels for each species: one using only the 19 climatic variables (climate submodel) and the other (geo-anthropic model) incorporating geophysical and anthropic variables, such as altitude, vegetation/land use maps, climate types, and NDVI. Each model was run independently in MaxEnt. The variables for each submodel were selected based on three criteria: high contribution values, permutation importance, and the results of the jackknife test. Jackknife analyses assess model gain under various hypotheses, including using all variables, each variable individually, and excluding each variable. A high gain with a single variable indicates that it significantly explains spatial variation on its own, while a decrease in gain without that variable suggests it is fundamentally important. The selected variables were then evaluated for multicollinearity using Pearson’s correlation (<0.7), retaining only non-correlated variables to avoid redundancy and maximize explanatory power. To eliminate correlated variables, additional runs were conducted using pairs of variables in MaxEnt, retaining the variable with the highest percentage contribution and permutation importance value (S1 Fig). All models were executed without hinge features, utilizing one bootstrap replicate and 50,000 background points. For each species, 70% of occurrences were used to calibrate the model, while 30% were reserved for validation. This approach was modified for *T. barberi*, which had a low number of occurrences (50% to calibrate/ 50% for validation). This iterative selection process identified the key bioclimatic and geographical factors most influential in distinguishing suitable from unsuitable habitats for the studied triatomine vectors.

### Modeling the potential distribution of Triatomine species

Potential distribution modeling was conducted using MaxEnt to identify habitat suitability for the triatomine vectors associated with the domestic cycle in Hidalgo. MaxEnt estimates distribution probabilities based on environmental characteristics (variables) at occurrence locations. It requires only presence records, as true absence data were unavailable. Instead, MaxEnt generates pseudo-absences to estimate probabilities [55,56]. The principle of maximum entropy involves restrictions placed on the model, which require the mean of each variable predicted by the model to be close to the average observed at presence locations. Among all potential distributions that meet these restrictions, MaxEnt selects the one with maximum entropy [55]. Using occurrence records and environmental variables, MaxEnt estimated an entropy value for each pixel, reflecting the statistical relationship between known distributions and habitat characteristics defined by bioclimatic and geographic variables [13]. Potential distribution maps ranged from 0 to 1, with values close to 0 indicating a null probability of presence and values approaching 1 signifying a high probability of presence. The final models for each vector were executed using the selected variables. Each model was run without hinge features, utilizing 50 bootstrap replicates and randomly assigning 30% of occurrences to test data for validation, along with 50,000 background points. The criteria were established after testing various parameters and selecting the most parsimonious ones based on an AUC value greater than 0.90. The final runs provided variable contributions, importance values, and jackknife analyses to rigorously assess model performance. The probability of presence maps delineated suitable habitat across environmental gradients in Hidalgo state.

### Statistical validation of potential distribution models

The potential distribution models were statistically validated using the area under the curve (AUC) metric. This metric was generated by both MaxEnt 3.4.1 [46], the software used to develop the species distribution models, and NicheToolbox, an additional modeling toolbox program [47]. The AUC assesses model performance based on the receiver operating characteristic (ROC) curve. The ROC curve plots the true positive rate (sensitivity) against the false positive rate (1 - specificity) across all possible probability thresholds for classifying presences. The AUC represents the area under this ROC curve, providing a measure of how effectively the model distinguishes between suitable habitat conditions for presence and unsuitable conditions. Higher AUC values indicate better discriminative ability of the model [57]. Sensitivity refers to the proportion of known presence locations classified as suitable habitat. Since true absence data are not available, specificity is estimated as “predicted fractional area” with MaxEnt [55]. Given the limitations of absence data, AUC in this context relies solely on sensitivity. AUC values range from 0 to 1, where 0 indicates no discriminative ability and 1 indicates perfect accuracy. A value near 0.5 suggests random performance. Common guidelines classify AUC values as follows: 0.50-0.60 (insufficient), 0.60-0.70 (poor), 0.70-0.80 (average), 0.80-0.90 (good), and 0.90-1.00 (excellent) [58]. Based on these frameworks, an AUC threshold of 0.8 was established to deem a model statistically robust, corresponding to good accuracy and reasonably skilled classification capabilities.

### Field validation of potential distribution models

The potential distribution models were validated through field efforts targeting vector collection within the domestic cycle (intra- and peri-domicile). Sampling sites were selected based on three criteria: 1) historical occurrence records [43], 2) sites without historical records (with at least one site per species), and 3) MaxEnt potential distribution maps developed in this study. Binary maps indicating potential presence and absence areas were created for each species. Due to a lack of theoretical or experimental data on the most suitable cutoff, we determined it based on MaxEnt suitability values. A pixel value of 0.5 suggests a 50% probability of presence, indicating nearly random likelihood, while a value of 1 indicates the highest probability. We classified pixels with values ≤0.75 as potential absence areas and those with values >0.75 as potential presence areas, accounting for any potential model overfitting. This cutoff allows for greater certainty in specimen collection for experimental validation. Sampling considered the known seasonality of each species from prior work [43]. For the seasonal *T. mexicana* triatomines, collections were performed by the laboratory staff in April-May 2022 and 2023 using three-night collection methods and mouse-baited traps in two localities, in addition to the standard man-hour search. The non-seasonal species *T. dimidiata* and the understudied *T. barberi* were sampled in August 2022 (three visits) and April 2023 (four visits) through standard man-hour searches conducted by the laboratory staff under the supervision of VCPH to maintain the same sampling effort and techniques used by the Secretary of Health, following the guidelines outlined in Official Mexican Standard NOM-032-SSA2–2014 [43]. Additional validation was obtained from the 2020–2021 records of the VCPH. This program employed the same standardized man-hour search protocol, reflecting consistent annual search efforts in the domestic cycle. Community participation enhanced the collection efforts. The collected specimens were identified to the species level using the keys by Lent & Wygodzinsky [59]. Trained staff in our laboratory identified our specimens, while specimens from the state program were identified at the state health laboratory of Hidalgo by trained personnel, both using the same identification keys.

### Determination of the sample size for the collection of Triatomines

A representative sample size was determined for the field validation based on the annual number of each vector species historically reported in the state. The sample size was calculated using a formula suitable for finite populations, as follows:


$$n=\frac{N*Z*p*q}{d^{2}*(N-1)+Z*p*q}$$ (1)

n = Sample size.

N = Population universe.

Z= Confidence level (1.96 with a confidence level of 95%).

d = Level of accuracy.

p = Percentage of population with desired attribute.

q = Percentage of the population that does not have the desired attribute.

By inputting these parameters, an appropriately sized sample was established for each vector species. This ensured the field collections would be representative and provide a robust test of the explanatory power of the models against observations from the target populations.

The accuracy of the MaxEnt model in classifying potential presence and absence sites was assessed by cross-validating it with experimental collection data targeted at the domestic cycle. Model assessment typically measures agreement between identified and observed classifications. Specifically, true positives, false positives, false negatives, and true negatives were calculated by comparing model classifications to validation observations (Table 1). In this study, the Kappa index was employed to assess concordance between the MaxEnt model outputs and field-derived presence/absence data for each species.

**Table 1:** Frequency table.

		Experimental validation		
		Presence	Absence	
Potential distribution model	Presence	A	B	pm = a + b
	Absence	C	D	am = c + d
		pv = a + c	av = b + d	N = a + b + c + d

The parameters of concordance and discrepancy used for evaluating the classification model are as follows: a = true positives; b = false positives; c = false negatives; d = true negatives; pm = presences in the model; am = absences in the model; pv = presences in the validation; av = absences in the validation; N = total

The Kappa index assesses overall agreement that accounts for chance [60]. It is calculated as:


$$k=\frac{P_{o}-P_{e}}{1-P_{e}}$$ (2)

Where *P*_*o*_ is the observed agreement ratio, calculated as (*a + d*)/*N* and *P*_*e*_ is the agreement expected by chance, given by *P*_*e*_ = (pm*pv + am*av)/ *N*^*2*^. A Kappa value of 1 indicates perfect agreement at sites classified as absence/presence in the model and the actual observations. Conversely, a Kappa value of 0 suggests that the observed agreement aligns with what would be expected by chance. The Kappa metric provides a quantitative measure of how effectively the MaxEnt distribution models classified validation locations compared to actual field observations of vector occurrences.

## Results

### Selected geoclimatic variables

The selection of relevant environmental variables (geo-climatic factors) is crucial for developing robust potential distribution models. Initially, 23 variables were considered based on literature highlighting ecological influences on vector studies [2,7,11,27,48–51]. We retained five variables for each triatomine species in the MaxEnt models, contributing at least 45% to importance and not correlated (Table 2). For *T. dimidiata*, climate types and vegetation/land uses were significant, while altitude and precipitation of the driest month (BIO14) were crucial. The model for *T. mexicana* included six variables, with climate types and altitude as the most influential. For *T. gerstaeckeri*, climate types and vegetation/land use were prominent, with altitude being most important. In contrast, *T. barberi* required five variables, where climate types and vegetation/land uses were significant, and precipitation of the driest quarter (BIO17) and isothermality (BIO3) were critical. Across the four species, climate types contributed the highest percentage (27.7-33.7%), followed by vegetation/land uses (17-31.5%). The most important variable varied: altitude for *T. dimidiata* (42.1%), *T. mexicana* (47.6%), and *T. gerstaeckeri* (77.9%), while precipitation of the driest quarter (BIO17) was dominant for *T. barberi* (51.1%). Pearson correlation analysis showed no correlation among variables, indicating strong explanatory power for the potential distribution models. In summary, climate types and altitude emerged as primary determinants of triatomine distributions across the studied species.

**Table 2:** Contribution- and importance-values of variables included in potential distribution models of triatomine species.

		*Triatoma dimidiata*	*Triatoma mexicana*	*Triatoma gerstaeckeri*	*Triatoma barberi*
Name	Variable	Contribution (%)			
Climate	Climate types	33.7	32.5	27.7	32.4
land_use	Vegetation and land use	21.9	17	24.1	31.5
Altitude	Altitude	3.9	25.3	22.1	–
Ndvi	Normalized Difference Vegetation Index (NDVI)	14.1	17.5	8.2	10.4
BIO14	Precipitation of Driest Month	19.3	–	–	–
BIO17	Precipitation of Driest Quarter	–	–	–	16
BIO6	Min Temperature of Coldest Month	–	–	15.7	–
BIO3	Isothermality (BIO2/BIO7) (×100)	7.1	–	–	9.7
BIO12	Annual Precipitation	–	4	–	–
BIO2	Mean Diurnal Range	–	3.7	–	–
BIO13	Precipitation of Wettest Month	–	–	2.2	–
BIO1	Annual Mean Temperature	–	–	–	–
BIO4	Temperature Seasonality (standard deviation ×100)	–	–	–	–
BIO5	Max Temperature of Warmest Month	–	–	–	–
BIO7	Temperature Annual Range (BIO5-BIO6)	–	–	–	–
BIO8	Mean Temperature of Wettest Quarter	–	–	–	–
BIO9	Mean Temperature of Driest Quarter	–	–	–	–
BIO10	Mean Temperature of Warmest Quarter	–	–	–	–
BIO11	Mean Temperature of Coldest Quarter	–	–	–	–
BIO15	Precipitation Seasonality (Coefficient of Variation)	–	–	–	–
BIO16	Precipitation of Wettest Quarter	–	–	–	–
BIO18	Precipitation of Warmest Quarter	–	–	–	–
BIO19	Precipitation of Coldest Quarter	–	–	–	–
Name	Variable	Importance (%)			
Altitude	Altitude	42.1	47.6	77.9	–
Ndvi	Normalized Difference Vegetation Index (NDVI)	10.9	16.1	5.6	17.5
land_use	Vegetation and land use	3.3	10.7	6.4	9.4
Climate	Climate types	2.7	4.8	5.5	1.7
BIO3	Isothermality (BIO2/BIO7) (×100)	1	–	–	20.3
BIO17	Precipitation of Driest Quarter	–	–	–	51.1
BIO14	Precipitation of Driest Month	40	–	–	–
BIO12	Annual Precipitation	–	17.4	–	–
BIO2	Mean Diurnal Range	–	3.4	–	–
BIO13	Precipitation of Wettest Month	–	–	2.5	–
BIO6	Min Temperature of Coldest Month	–	–	2.1	–
BIO1	Annual Mean Temperature	–	–	–	–
BIO4	Temperature Seasonality (standard deviation ×100)	–	–	–	–
BIO5	Max Temperature of Warmest Month	–	–	–	–
BIO7	Temperature Annual Range (BIO5-BIO6)	–	–	–	–
BIO8	Mean Temperature of Wettest Quarter	–	–	–	–
BIO9	Mean Temperature of Driest Quarter	–	–	–	–
BIO10	Mean Temperature of Warmest Quarter	–	–	–	–
BIO11	Mean Temperature of Coldest Quarter	–	–	–	–
BIO15	Precipitation Seasonality (Coefficient of Variation)	–	–	–	–
BIO16	Precipitation of Wettest Quarter	–	–	–	–
BIO18	Precipitation of Warmest Quarter	–	–	–	–
BIO19	Precipitation of Coldest Quarter	–	–	–	–

### Potential distribution modeling

#### Significance of variables obtained with the jackknife test.

**Fig 1:**
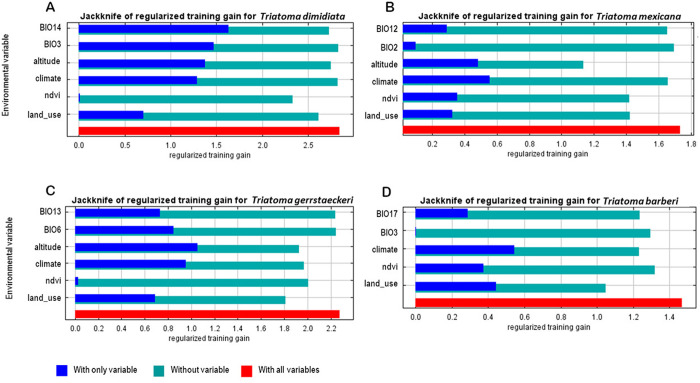
Jackknife test results obtained with MaxEnt for (A) *T. dimidiata*, (B) *T. mexicana*, (C) *T. gerstaeckeri* and (D) *T. barberi.* The navy-blue bar represents the gain from the model only with the variable. The turquoise bar represents the gain of the model if the variable is removed. The red bar represents the model gain with all variables included.

We used the jackknife test to assess each variable’s impact on the potential distribution models (Fig 1). For *T. dimidiata*, precipitation of the driest month (BIO14) explained over half the variation, followed by isothermality (BIO3) and altitude. In *T. mexicana*, climate types were the main contributor, with altitude next. For *T. gerstaeckeri*, altitude accounted for most variation, followed by climate types. In *T. barberi*, climate types of lead, followed by vegetation/land uses. Key variables whose exclusion significantly affected model performance included NDVI and vegetation/land uses for *T. dimidiata* and *T. mexicana*, and altitude and climate types for *T. gerstaeckeri*. In summary, climate types were most significant for *T. mexicana* and *T. barberi*, while altitude and BIO14 were primary for *T. dimidiata* and *T. gerstaeckeri*. Excluding NDVI and altitude harmed models for *T. dimidiata* and *T. mexicana*, while omitting vegetation/land uses affected *T. gerstaeckeri* and *T. barberi*.

### Potential distribution maps (probability of presence)

**Fig 2:**
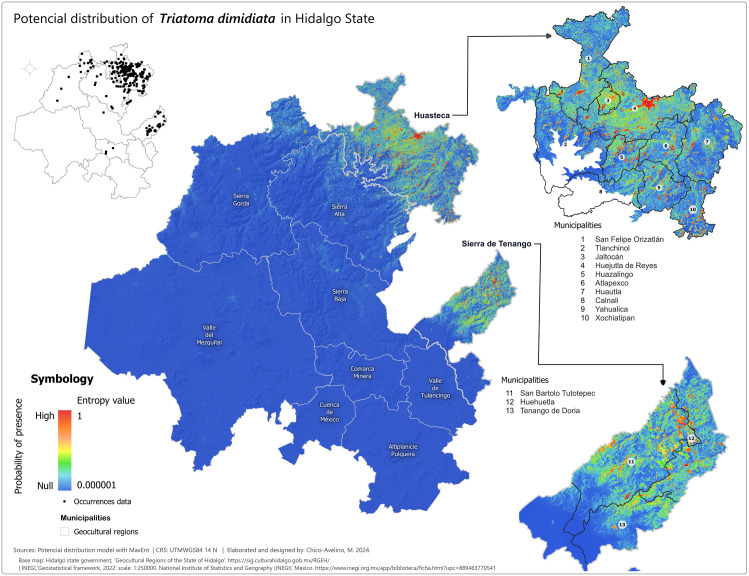
Potential distribution model of *T. dimidiata* in the state of Hidalgo. Colder tones represent the lowest presence probabilities (0.000001) while warmer shades represent the highest values (1). Municipal boundaries are delineated with black borders and identified with white circles containing the municipality name and number listed beside each enlarged section. Lighter grey lines demarcate the geocultural regions. Black dots signify species occurrence records used to train the MaxEnt model. Base map: Hidalgo State Government, Geocultural Regions of the State of Hidalgo (open access). [https://sig.culturahidalgo.gob.mx/RGEH/] | INEGI,‘Geostatistical framework, 2022’ scale: 1:250000. National Institute of Statistics and Geography (INEGI). Mexico. [https://www.inegi.org.mx/app/biblioteca/ficha.html?upc=889463770541]. This information is also included at the bottom of the map.

**Fig 3:**
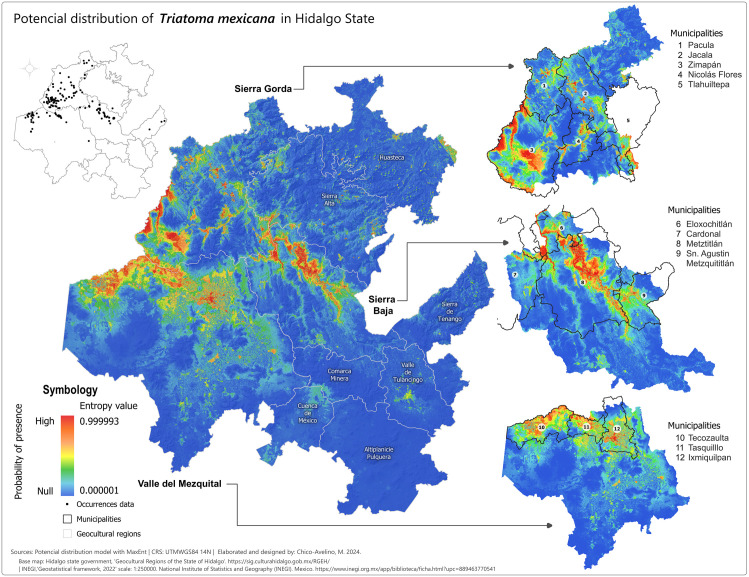
Potential distribution model of *T. mexicana* in the state of Hidalgo. Colder tones represent the lowest presence probabilities (0.000001) while warmer shades represent the highest values (0.999993). Municipal boundaries are delineated with black borders and identified with white circles containing the municipality name and number listed beside each enlarged section. Lighter grey lines demarcate the geocultural regions. Black dots signify species occurrence records used to train the MaxEnt model. Base map: Hidalgo State Government, Geocultural Regions of the State of Hidalgo (open access). [https://sig.culturahidalgo.gob.mx/RGEH/] | INEGI,‘Geostatistical framework, 2022’ scale: 1:250000. National Institute of Statistics and Geography (INEGI). Mexico. [https://www.inegi.org.mx/app/biblioteca/ficha.html?upc=889463770541]. This information is also included at the bottom of the map.

**Fig 4:**
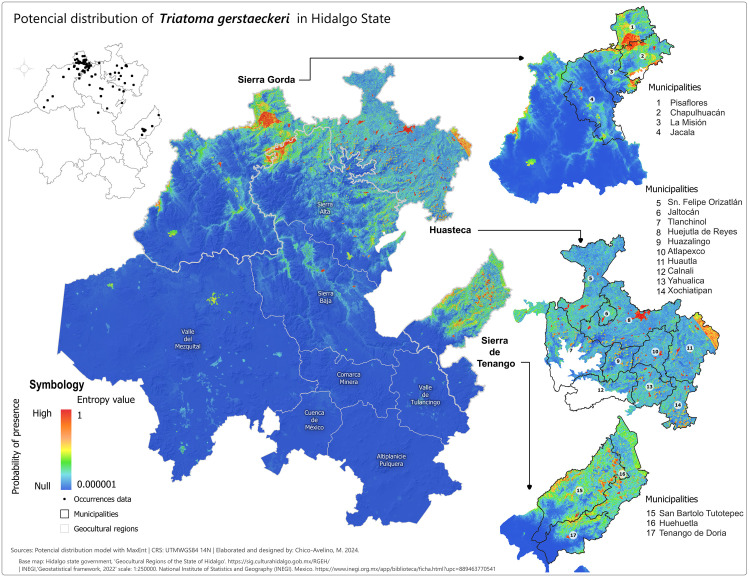
Potential distribution model of *T. gerstaeckeri* in the state of Hidalgo. Colder tones represent the lowest presence probabilities (0.000001) while warmer shades represent the highest values (1). Municipal boundaries are delineated with black borders and identified with white circles containing the municipality name and number listed beside each enlarged section. Lighter grey lines demarcate the geocultural regions. Black dots signify species occurrence records used to train the MaxEnt model. Base map: Hidalgo State Government, Geocultural Regions of the State of Hidalgo (open access). [https://sig.culturahidalgo.gob.mx/RGEH/] | INEGI,‘Geostatistical framework, 2022’ scale: 1:250000. National Institute of Statistics and Geography (INEGI). Mexico. [https://www.inegi.org.mx/app/biblioteca/ficha.html?upc=889463770541]. This information is also included at the bottom of the map.

**Fig 5:**
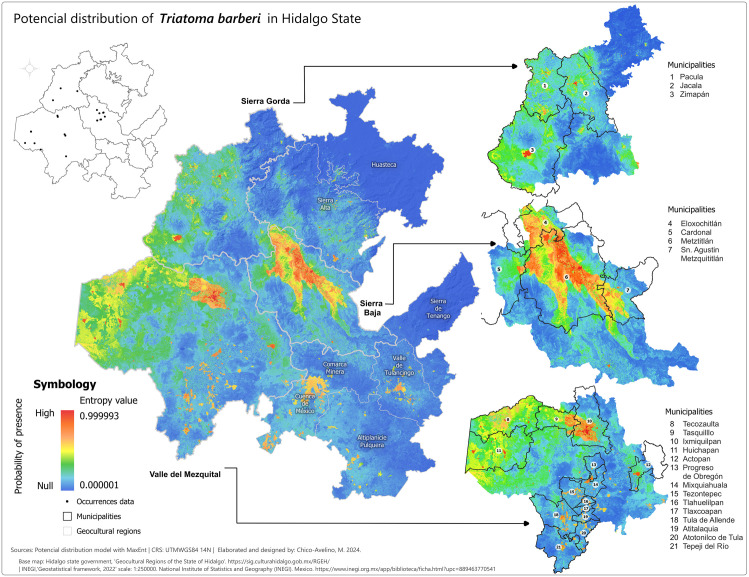
Potential distribution model of *T. barberi* in the state of Hidalgo. Colder tones represent the lowest presence probabilities (0.000001) while warmer shades represent the highest values (0.999993). Municipal boundaries are delineated with black borders and identified with white circles containing the municipality name and number listed beside each enlarged section. Lighter grey lines demarcate the geocultural regions. Black dots signify species occurrence records used to train the MaxEnt model. Base map: Hidalgo State Government, Geocultural Regions of the State of Hidalgo (open access). [https://sig.culturahidalgo.gob.mx/RGEH/] | INEGI,‘Geostatistical framework, 2022’ scale: 1:250000. National Institute of Statistics and Geography (INEGI). Mexico. [https://www.inegi.org.mx/app/biblioteca/ficha.html?upc=889463770541]. This information is also included at the bottom of the map.

After selecting the appropriate variables, we created potential distribution maps for each species using MaxEnt presence probability layers. For *T. dimidiata* (Fig 2), the highest probabilities (0.75-1) were concentrated in the eastern and northeastern parts of Hidalgo, especially in the Sierra de Tenango and Huasteca regions, including municipalities like San Bartolo Tutotepec and Huejutla. In other regions, the presence probabilities for *T. dimidiata* were negligible. For *T. mexicana* (Fig 3), high probabilities (0.75-0.999993) were found in central, western, and northwestern Hidalgo, particularly in the Sierra Baja, with key areas including Eloxochitlán, Metztitlán and Tecozautla. The *T. gerstaeckeri* model (Fig 4) indicated high presence probabilities in the eastern, northeastern and northern regions, particularly in the Sierra de Tenango, Huasteca and Sierra Gorda, with notable municipalities such as San Bartolo Tutotepec, Huejutla de Reyes and Pisaflores. In contrast, low presence probabilities were assigned to areas like the Valle del Mezquital and Comarca Minera. The *T. barberi* model (Fig 5) showed a broader distribution, with high probabilities (0.75-0.999993) in the northwest Sierra Gorda and central Sierra Baja, including municipalities like Zimapán and Metztitlán. The Valle del Mezquital also had several suitable municipalities. Lower probabilities were detected in areas like Pachuca in the Comarca Minera and Tizayuca in Cuenca de México, with no presence in the Huasteca and Sierra de Tenango. These results reflect a differential distribution consistent with prior analyses [43].

### Statistical validation of potential distribution models

The statistical validity of the potential distribution models was assessed using the AUC metric from MaxEnt (S4 Fig) and NicheToolBox. All four models achieved AUC values greater than 0.90, indicating over 90% classification of occurrences as potential presence sites. Specifically, *T. dimidiata* had an AUC of 0.969 with MaxEnt and 0.983 with NicheToolBox. *T. mexicana* achieved 0.935 (MaxEnt) and 0.969 (NicheToolBox). For *T. gerstaeckeri*, the AUC was 0.957 (MaxEnt) and 0.966 (NicheToolBox). *T. barberi* had AUC values of 0.94 (MaxEnt) and 0.945 (NicheToolBox). These results indicate strong statistical performance for all four triatomine species, with AUC values consistently above 90%.

### Field validation of potential distribution models

**Fig 6:**
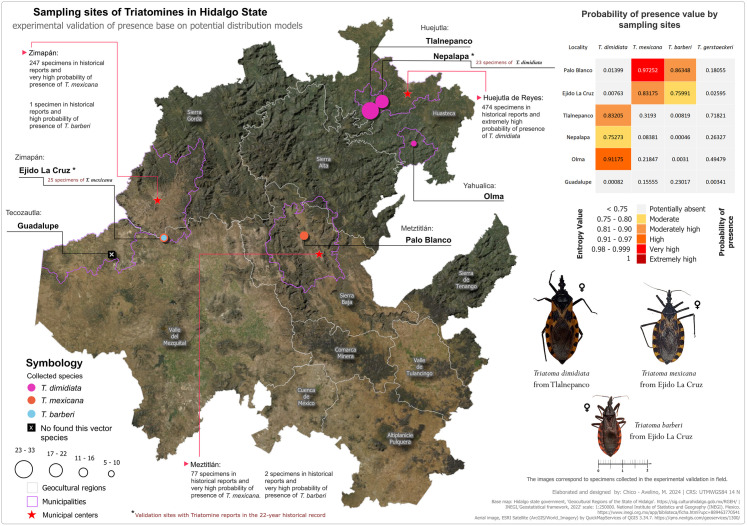
Maps field validation sites of the domestic cycle for *T. dimidiata*, *T. mexicana* and *T. barberi* in Hidalgo state. Validation sites are displayed in bold text. Colored dots locale collections for *T. dimidiata* (in pink), *T. mexicana* (in orange) and *T. barberi* (sky blue). Circle size proportionate specimen count. Municipal centers marked with red stars list historical records and species probabilities. The negative control site is outlined with a white “X”. A table provides probability ranges for selected sites, shaded from light gray (<0.75) to dark red (1). Municipal boundaries appear as purple lines and geocultural regions in light grey. The sites marked with an asterisk are the only ones with prior records. Photographs of representative specimens validate the identification of vector species collected during field validation. Aerial image, ESRI Satellite (ArcGIS/World_Imagery) by QuickMapServices of QGIS 3.34.7. (open access). [https://qms.nextgis.com/geoservices/1300/]. Base map: Hidalgo State Government, Geocultural Regions of the State of Hidalgo (open access). [https://sig.culturahidalgo.gob.mx/RGEH/] | INEGI,‘Geostatistical framework, 2022’ scale: 1:250000. National Institute of Statistics and Geography (INEGI). Mexico. [https://www.inegi.org.mx/app/biblioteca/ficha.html?upc=889463770541]. This information is also included at the bottom of the map.

To validate the potential distribution results, we conducted field validation (Fig 6 and Table 3) at six locations: two with known triatomine records (Nepalapa and La Cruz) and four identified as potential habitats (Tlalnepanco, Olma and Palo Blanco). Fig 6 shows the sites for validating the presence of the four vector species in municipalities of Huejutla (Huasteca), Yahualica (Huasteca), Metztitlán (Sierra Baja), and Zimapán and Tecozautla (Valle del Mezquital). In Huejutla de Reyes, known for a high historical record of *T. dimidiata* (474 specimens), we visited Tlalnepanco, Nepalapa, and Olma, collecting a total of 65 *T. dimidiata* specimens, confirming its high presence probability. We validated the expected absence of *T. mexicana*, *T. gerstaeckeri*, and *T. barberi*, as predicted by the models. In Metztitlán and Zimapán, we confirmed *T. mexicana*’s presence by collecting specimens from Palo Blanco and La Cruz. Most specimens were found peridomiciliary, consistent with *T. mexicana*’s nocturnal habits. We found one adult female and four nymphs of *T. barberi* in Zimapán, confirming its presence despite limited historical records. However, we could not validate *T. gerstaeckeri* due to security issues, but its absence was corroborated at sites with very low model probability. As a negative control, we visited Guadalupe, where no specimens of any of the four species were found, thereby confirming their absence mapping. We inspected 82 out of 700 dwellings (11.7%), accessing 18 houses (2.5% of total dwellings) that all contained triatomines, yielding 97 specimens, mostly intradomiciliary (86/97, 89.7%). Our collection strategies were effective, particularly for *T. dimidiata* using the man-hour technique, while community participation helped with *T. barberi* and *T. mexicana,* aligning with their nocturnal behavior [43]. These findings demonstrate that our approach effectively validated the potential distributions of *T. dimidiata*, *T. mexicana,* and *T. barberi*. While we could not validate *T. gerstaeckeri*, the results suggest that its model would also perform well, warranting further investigation.

**Table 3:** Number of specimens collected of *T. dimidiata*, *T. mexicana* and *T. barberi* in the experimental validation.

Principal municipality	Locality	Geocultural Regions	Potentially absent species	Potentially present species	Total houses	Total of houses visited	Positive houses	Specimens	*T. mexicana* (MASR = 7)			*T. dimidiata* (MASR = 8)			*T. barberi* (MASR = 4)			Environment		Sampling type			
									F	M	N	F	M	N	F	M	N	I	P	Cc	Mc	M-H	T
Metztlitlán	Palo Blanco	Sierra Baja	*T. dimidiata* *T. gerstaeckeri*	*T. mexicana* *T. barberi*	15	6	4	16	7	8	1	0	0	0	0	0	0	10	6	14	2	0	NA
Zimapán	Ejido La Cruz	Valle del Mezquital	*T. dimidiata* *T. gerstaeckeri*	*T. mexicana* *T. barberi*	62	10	4	16	5	6	0	0	0	0	1	0	4	11	5	14	1	0	1
Huejutla de Reyes	Tlalnepanco	Huasteca	*T. gerstaeckeri T. mexicana* *T. barberi*	*T. dimidiata*	317	36	5	33	0	0	0	14	6	13	0	0	0	33	0	0	0	33	NA
	Nepalapa	Huasteca	*T. gerstaeckeri T. mexicana* *T. barberi*	*T. dimidiata*	41	21	2	22	0	0	0	6	4	12	0	0	0	22	0	0	0	22	NA
Yahualica	Olma	Huasteca	*T. gerstaeckeri T. mexicana* *T. barberi*	*T. dimidiata*	45	5	3	10	0	0	0	5	0	5	0	0	0	10	0	0	0	10	NA
Tecozautla	Guadalupe	Valle del Mezquital	*T. dimidiata* *T. mexicana* *T. gerstaeckeri T. barberi*	NA	220	4	0	0	0	0	0	0	0	0	0	0	0	0	0	0	0	0	0
				**Total**	700	82	18	97	12	14	1	25	10	30	1	0	4	86	11	28	3	65	1

MASR: Minimum annual samples required; NA: Not applicable; F: Female; M: Male; N: Nymphs; I: Intradomicile; P: Peridomicile; Cc: Community collection; Mc: Manual collection; M-H: Man-Hour; T: Trap

### Sample size for collected Triatomines

**Fig 7:**
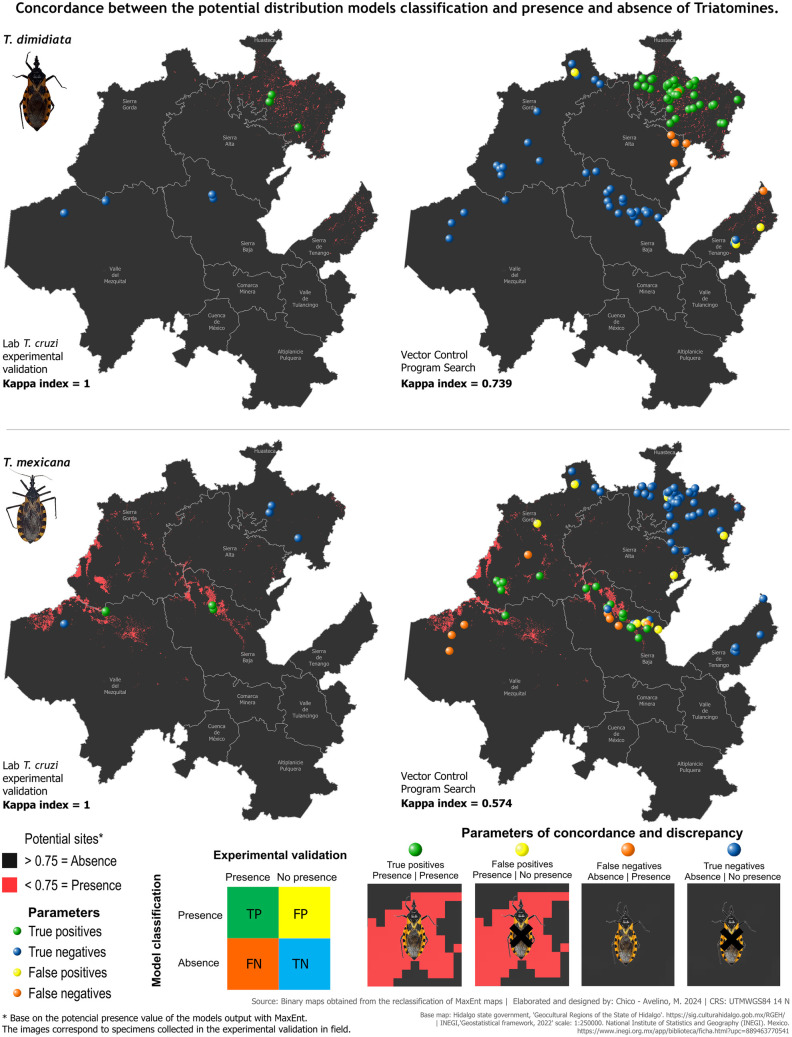
Concordance between presence/absence classifications from *T. dimidiata* and *T. mexicana* potential distribution models against field validation data targeted to the domestic cycle. The background map depicts MaxEnt model probability representation of presence (red) and absence (dark gray). Data points show locations from our validation and Vector Control Program of the state of Hidalgo surveys. Colors represent concordance and discrepancy parameters of true positives (green), true negatives (blue), false positives (yellow) and false negatives (orange). The color ramp table exemplifies these parameters. Spatially overlaying the points on the model classification visually illustrates where agreements and mismatches occurred between inferences and empirical findings. Images depicting agreement and discrepancy were captured by zooming in on potential presence and absence sites from the binary maps, while vector species images were taken from specimens collected during field validation. Base map: Hidalgo State Government, Geocultural Regions of the State of Hidalgo (open access). [https://sig.culturahidalgo.gob.mx/RGEH/] | INEGI,‘Geostatistical framework, 2022’ scale: 1:250000. National Institute of Statistics and Geography (INEGI). Mexico. [https://www.inegi.org.mx/app/biblioteca/ficha.html?upc=889463770541]. This information is also included at the bottom of the map.

**Fig 8:**
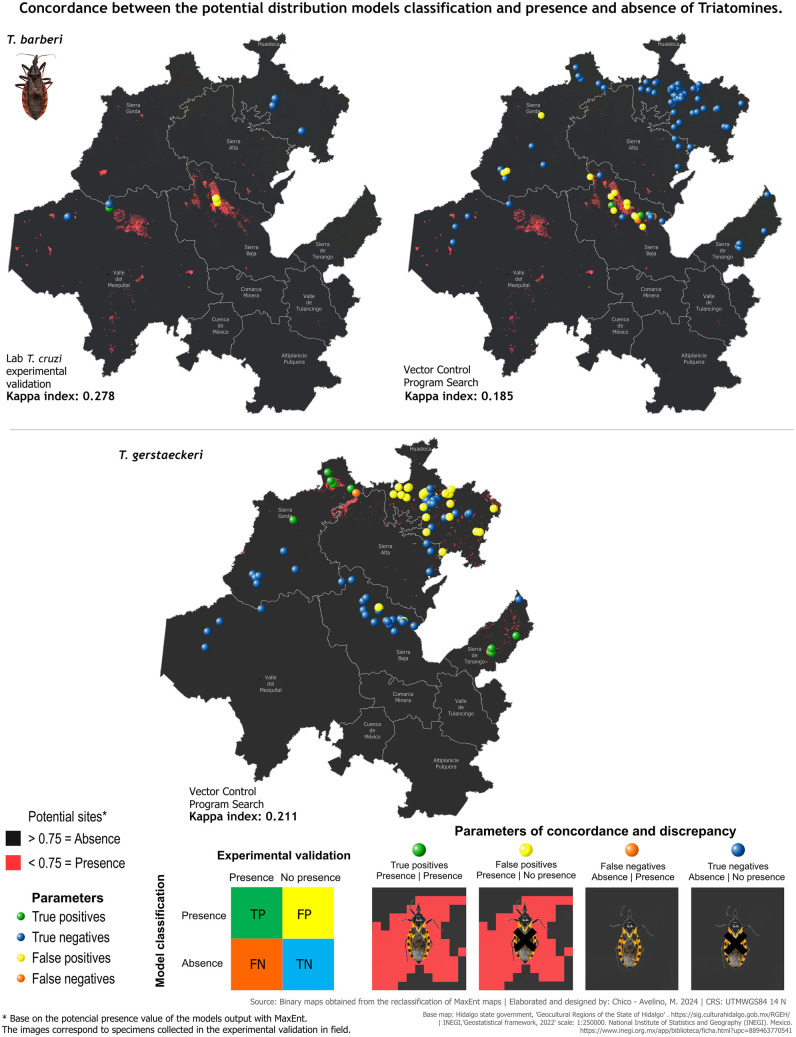
Concordance between predicted presence/absence classifications from *T. barberi* and *T. gerstaeckeri* potential distribution models against field validation data targeted to the domestic cycle. The background map depicts MaxEnt model probability mapping of presence (red) and absence (dark gray). Data points show locations from our validation and/ or by Vector Control Program of the state of Hidalgo surveys. Colors represent concordance and discrepancy parameters of true positives (green), true negatives (blue), false positives (yellow) and false negatives (orange). The color ramp table exemplifies these parameters. Spatially overlaying the points on the model classification visually illustrates where agreements and mismatches occurred between inferences and empirical findings. Images depicting agreement and discrepancy were captured by zooming in on potential presence and absence sites from the binary maps, while vector species images were taken from specimens collected during field validation. Base map: Hidalgo State Government, Geocultural Regions of the State of Hidalgo (open access). [https://sig.culturahidalgo.gob.mx/RGEH/] | INEGI,‘Geostatistical framework, 2022’ scale: 1:250000. National Institute of Statistics and Geography (INEGI). Mexico. [https://www.inegi.org.mx/app/biblioteca/ficha.html?upc=889463770541]. This information is also included at the bottom of the map.

Sample size calculations ensured statistically representative collections of triatomines during experimental validation. We used historical records [43] to determine the following: average annual specimen counts (99 for *T. dimidiata*, 62 for *T. mexicana*, 20 for *T. gerstaeckeri*, 6 for *T. barberi*), years of records (20 for *T. dimidiata*, 18 for *T. mexicana* and *T. gerstaeckeri*, 12 for *T. barberi*), and total specimen records over 22 years (1975 for *T. dimidiata*, 1106 for *T. mexicana*, 334 for *T. gerstaeckeri*, 62 for *T. barberi*). Minimum annual samples required were 8 for *T. dimidiata*, 7 for *T. mexicana*, 6 for *T. gerstaeckeri*, and 4 for *T. barberi*. We collected 65 *T. dimidiata*, 27 *T. mexicana*, and 5 *T. barberi*, exceeding these requirements (Table 3). To evaluate model accuracy, we compared predicted presence/absence with our field data from six sites and annual data from 100 localities by VCPH, for the same year. For *T. dimidiata*, our validation achieved 100% concordance (Kappa = 1), with true positives in the Huasteca region and true negatives elsewhere (Fig 7). No false positives or negatives occurred. In contrast, the VCPH concordance was lower (Kappa = 0.739), with false positives and negatives primarily in the Huasteca region, east of Sierra Alta, east of Sierra de Tenango, and north of Sierra Gorda. For *T. mexicana*, we again achieved 100% concordance (Kappa = 1), with true positives in Sierra Baja and Valle del Mezquital, and true negatives elsewhere (Fig 7). The VCPH showed lower concordance (Kappa = 0.574), with false positives in multiple regions (Huasteca, Sierra Gorda, and Sierra Baja) and false negatives in Sierra Gorda, Sierra Baja, and Valle del Mezquital. The *T. barberi* validation revealed low concordance (Kappa = 0.278), with true negatives in Huasteca and Valle del Mezquital, one true positive in Valle del Mezquital (Ejido La Cruz), and false positives in Sierra Baja (Fig 8). VCPH concordance was even lower (Kappa = 0.185), showing true negatives in Huasteca, Sierra Gorda, Sierra Baja, Valle del Mezquital, and Sierra de Tenango, with a true positive in the Sierra Baja region and discrepancies in both Sierra Baja and Sierra Gorda. For *T. gerstaeckeri*, we could only analyze VCPH data, which indicated low concordance (Kappa = 0.211), with true positives in the northern Sierra Gorda and Sierra de Tenango, true negatives in Huasteca, Sierra Alta, Sierra Baja, south of Sierra Gorda, and Valle del Mezquital, alongside false positives in Huasteca and Sierra Baja and false negatives in the northern Sierra Gorda and Sierra de Tenango (Fig 8). In summary, the models for *T. dimidiata* and *T. mexicana* demonstrated strong classification capabilities. Targeted collections under optimized conditions could further improve validation concordance. Additionally, experimental mapping for *T. gerstaeckeri* and expanding records for *T. barberi* are priorities to enhance their presence modeling.

## Discussion

The distribution of species is influenced by various historical, ecological, and physiological factors that vary geographically. These species encounter diverse biotic and abiotic conditions, which subject their populations to different selection pressures [61]. Potential distribution models are commonly used to map the ranges of vectors and identify the environmental factors affecting their geographical distributions. Previous studies have effectively utilized MaxEnt to analyze habitat suitability and transmission risk patterns for various disease vectors. In this study, we focused on the domestic cycle of triatomines and developed potential distribution models using MaxEnt for four dominant vectors of Chagas disease (*T. dimidiata*, *T. mexicana*, *T. gerstaeckeri*, and *T. barberi*) in Hidalgo, Mexico. These models were based on occurrence records, climate, topography, and land use data. By integrating the models with field collections, we achieved statistical validation and the first experimental ground-truthing of modeled vector presence/absence for Chagas disease in domestic settings. Our analysis aims to provide potential distribution maps informed by real-world testing, enhancing the understanding of niche determinants for these species and supporting evidence-based surveillance and control decisions. Our analysis confirmed several important environmental factors that influence the distribution of Chagas disease vectors [7,27,62,63]. Variables related to climate, topography, vegetation, and land use effectively characterized the potential habitats of the four triatomine species. Findings were consistent with prior research, highlighting the significant role of climate types across models. For instance, *T. dimidiata* showed the highest probability of presence in humid subtropical climate zones, aligning with its known associations in Hidalgo and other Mexican states [43,64–68]. *T. mexicana* thrived in semi-arid subtropical and semi-arid climates, consistent with previous studies [29,43]. *T. gerstaeckeri* was most probable in hot subhumid and humid subtropical zones, while *T. barberi* was classified as having the highest likelihood of presence in semi-arid warm and semi-dry warm climates, reflecting its correlation with dry conditions in Hidalgo and other states [29,43,69–71]. These observations suggest that the vectors’ ability to withstand varying environmental conditions, including drought tolerance, influences their ecological niches [72]. Vegetation and land use also played significant roles, with some species, like *T. dimidiata* and *T. barberi*, confined to domestic habitats, while others, such as *T. mexicana* and *T. gerstaeckeri*, utilized diverse, less modified vegetation. This aligns with previous findings on varying degrees of anthropic adaptation and seasonal patterns [7,11]. Altitude was another critical factor, affecting most species’ distributions [26,64]. However, limited data may have hindered the detection of altitude effects for *T. barberi*, highlighting the need for further observations. Given that altitude impacts *T. cruzi* virulence in triatomines like *T. dimidiata* [73], further research on the interplay between geoclimatic conditions, vector ecology, and parasite infectivity could inform more targeted control strategies. Precipitation also emerged as a crucial factor for certain species, corroborating prior global modeling [7]. Variations in microclimates, such as those found in domestic settings, can significantly affect vector populations due to fluctuations in humidity and temperature [74]. For example, drought impacts *T. brasiliensis* populations in various environments, with population fluctuations tied to drought seasonality [75]. Our observations of *T. mexicana* in Hidalgo revealed seasonality linked to drought and rainy seasons, while *T. dimidiata* appeared unaffected by seasonality, indicating that microclimate conditions influence domestic species differently than visiting species. Monitoring fine-scale domestic and peri-domestic microclimates could elucidate patterns and seasonality. Understanding the relationship between climatic factors and the physiology of vectors is essential for interpreting distribution patterns. Research integrating distribution models with physiological thermal tolerances has shown that species with greater tolerance to low temperatures generally have wider latitudinal ranges [25]. Thus, evaluating the physiological characteristics of species distributed in Hidalgo will be crucial. Our findings validate known macroclimate and landscape determinants, but future work incorporating microclimate data may clarify local drivers and address anomalies in some variables. Continued refinement will enhance the characterization of vector-environment interactions, which is vital for developing evidence-based control strategies.

The four vector species exhibited distinct potential distributions, aligning with previous analyses [43]. The potential distribution models accurately captured the known distribution patterns of these species, validating the effectiveness of MaxEnt for mapping Chagas disease vectors in domestic settings in Hidalgo. Notably, while *T. barberi* demonstrated the widest potential distribution, *T. dimidiata* and *T. mexicana* were more localized. These results have significant implications for surveillance and control efforts, suggesting that priority regions, such as Metztitlán for *T. mexicana*, should be targeted for interventions.

Our models differ from previous studies on Chagas disease vectors in Mexico, which often used environmental predictors with a spatial resolution of approximately 1 km [26,28,29,76]. Our focus on the domestic cycle of triatomines in Hidalgo necessitated a finer scale (60 m), allowing for more accurate characterization of potential presence/absence sites closely aligned with domestic environments. This approach supports the identification of specific housing conditions that provide particular niches for vector species [35,77]. The produced maps offer spatially explicit guidance for targeted field activities and interventions. This focused approach enables more efficient, evidence-based public health programs. However, there is still potential for model refinement by incorporating additional occurrence records, especially from under-sampled areas like parts of the Valle del Mezquital. Comparing model predictions with reported Chagas disease incidence could further validate these maps for assessing regional transmission risk. Long-term entomological and serological monitoring aligned with model forecasts will be critical for evaluating performance as environmental conditions change and capturing shifts in vector distributions.

Rigorous validation assessed the models’ classification capabilities using the AUC metric with MaxEnt and NicheToolBox, all achieving high AUC values over 0.9, indicating excellent classification of known presence locations. We implemented experimental validation through field sampling targeted at probable presence/absence sites within the domestic cycle. This real-world validation tested model applicability under operational conditions, bolstered by collection records from the same years (2020–2021) provided by the Hidalgo State Vector Control program. The Kappa index and AUC quantitatively assessed the agreement between models and observations, demonstrating high concordance and classification precision. For validating *T. dimidiata* and *T. mexicana*, a presence/absence threshold of 0.75 proved effective, but exploring additional thresholds for generating binary maps of Chagas disease vectors at different scales could be beneficial, as varying MaxEnt thresholds can yield different evaluation metrics [78]. This integrated approach of potential distribution modeling, statistical, and experimental validation combined analytical and empirical methods, enhancing mapping reliability. Field validation helped identify the most efficient collection techniques for each vector species, confirming the real presence of three Chagas disease vectors in the domestic cycle in Hidalgo. Overall, validation reinforced that MaxEnt models generate reliable potential distributions, accurately reflecting each vector’s niche, thereby supporting entomological surveillance, intervention planning, and transmission risk evaluation for evidence-based control strategies.

One concern regarding potential distribution models is their explanatory and predictive capabilities, which can be affected by biases in occurrence data from varied and non-systematic sources. A preprocessing step is recommended to avoid data clustering and over-occurrence that could impact modeling accuracy [79,80]. The quality of the information layers used for modeling—considering their temporal and spatial scales—also influences the delineation of potential areas. Implementing rigorous design approaches and utilizing tools that automate the selection of optimal parameters can enhance model certainty and reproducibility [81,82]. It has been suggested to utilize platforms that integrate a variety of modeling approaches to enhance the reliability of the models [83]. Additionally, the creation of consensus maps to identify potential sites classified by different models has been reported for Chagas disease vector species [2]. However, this aspect falls outside the scope of the current study. While our MaxEnt models performed well, discussions about parameterization biases in this package exist [84,85]. However, we think our model design criteria were adequate, supported by statistical and experimental validation, and successful specimen collection. Nonetheless, there is room for improvement in refining models to enhance their application in controlling Chagas disease vectors.

Potential distribution models are crucial for public health efforts against Chagas disease, identifying regions with the highest transmission risk. By analyzing environmental, ecological, and socioeconomic factors, these models enable public health officials to prioritize interventions and allocate resources effectively. Understanding the geographic distribution of disease vectors also enhances outbreak monitoring and prediction, allowing timely responses to protect vulnerable populations. The models developed in this study could inform more effective public health strategies in Hidalgo, thereby strengthening efforts to prevent transmission and safeguard communities at risk of Chagas disease.

## Conclusion

This study developed potential distribution models for four major Chagas disease vectors in Hidalgo, Mexico, using MaxEnt modeling with robust occurrence datasets and environmental variables. Statistical validation showed good to excellent discrimination, while experimental validation through field collections confirmed high agreement between observed and predicted presence/absence. These models can assist public health authorities in prioritizing surveillance and interventions in areas of high vector presence, potentially interrupting transmission dynamics. However, limitations related to environmental variables and data quality exist, and future research should incorporate additional factors and expand validation efforts. Overall, these validated models will enhance public health strategies against Chagas disease in Hidalgo, aiding in the prevention of transmission and protection of at-risk communities.

## Supporting information

S1 Fig Selection process for variables with high explanatory power in potential distribution models of Triatomine species using MaxEnt. A) Shows the raster layers of the 19 climatic variables used in the climatic submodel. B) Displays the raster layers of the 4 geophysical variables in the geo-anthropic submodel. Both graphs illustrate the AUC for each submodel, with 70% of occurrence data for calibration (Train Data) and 30% for validation (Train test). C) Presents two criteria for selecting submodel variables: contribution and importance values (Table) and jackknife (graph); green-marked variables were selected. D) Illustrates the third selection criterion using the Pearson method, where green indicates strong correlation, yellow indicates weak correlation, and red indicates no correlation. E) Shows contribution and importance values from independent runs of correlated variable pairs (phase D) with MaxEnt, selecting the variable with the highest values, such as BIO14. F) Represents the final model with the 6 most explanatory variables for *T. dimidiata*, along with the results from MaxEnt: potential distribution map, AUC, and jackknife.
(TIF)

S2 Fig Pearson variation coefficient values evaluated for the variables included in the final potential distribution models of Triatomine species. Each plot shows the Pearson correlation analysis of the final variables for each species, with correlation values in the cells. The color gradient indicates correlation strength: gray for no correlation (near zero), blue purple for strong positive correlation (one), and neon pink for strong negative correlation (-1).
(TIF)

S3 Fig Map of the municipal division and geocultural regions of the state of Hidalgo. Municipalities are represented with a black border and an identifier number and are listed alphabetically on the right side of the map. The areas that comprise the geocultural regions that are represented in colors; in light fiusha color the Huasteca region, in light olive green the Sierra Gorda, in light blue the Sierra Alta, in sand color the Sierra Baja, in lilac color the Sierra de Tenango, in aqua green the Valle del Mezquital, light pink the Comarca Minera, the Cuenca de México in yellow, the Valle de Tulancingo in light orange and the Altiplanicie pulquera in light gray. Base map: Hidalgo State Government, Geocultural Regions of the State of Hidalgo (open access). [https://sig.culturahidalgo.gob.mx/RGEH/] | INEGI,‘Geostatistical framework, 2022’ scale: 1:250000. National Institute of Statistics and Geography (INEGI). Mexico. [https://www.inegi.org.mx/app/biblioteca/ficha.html?upc=889463770541]. This information is also included at the bottom of the map.
(TIF)

S4 Fig Receiver Operating Characteristic (ROC) curve obtained with MaxEnt for A) *T. dimidiata*, B) *T. mexicana*, C) *T. gerstaeckeri* and D) *T. barberi.* The red line represents the trend of the mean of the AUC values obtained in the 50 model replicates. The navy-blue area is the mean + /- one standard deviation of the AUC values obtained in the 50 model replicates. The black line corresponds to a random model that would have an AUC value of 0.5.
(TIF)
