# Supplementary figures and images for: MaxEnt modeling and risk evaluation of chagas disease vectors in the domestic cycle of Hidalgo, Mexico
Source: PLoS Negl Trop Dis. 2025 Jul 31;19(7):e0013199. doi: 10.1371/journal.pntd.0013199 (PMC12312938; doi:10.1371/journal.pntd.0013199)

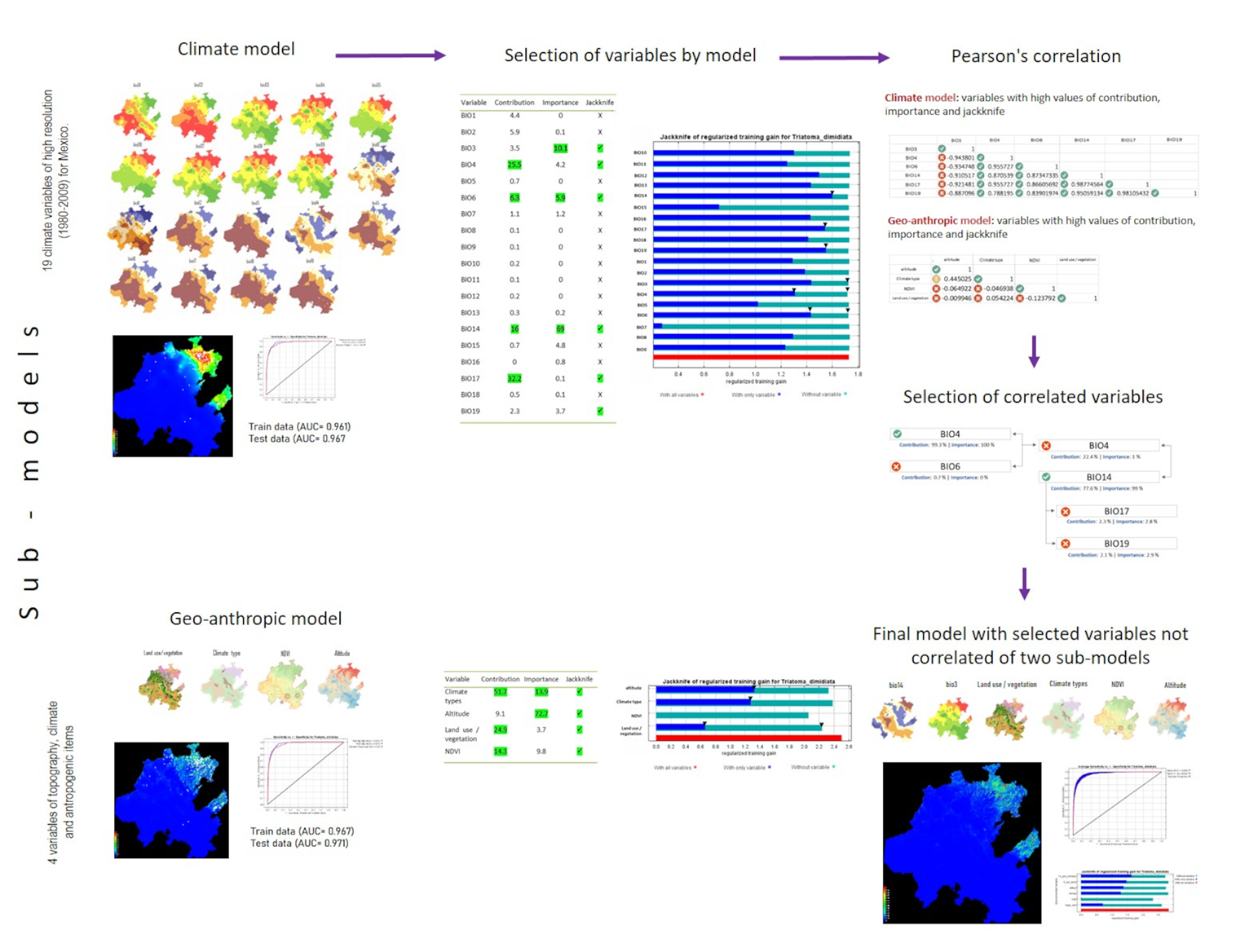

Supplement: S1 Fig — A) Shows the raster layers of the 19 climatic variables used in the climatic submodel. B) Displays the raster layers of the 4 geophysical variables in the geo-anthropic submodel. Both graphs illustrate the AUC for each submodel, with 70% of occurrence data for calibration (Train Data) and 30% for validation (Train test). C) Presents two criteria for selecting submodel variables: contribution and importance values (Table) and jackknife (graph); green-marked variables were selected. D) Illustrates the third selection criterion using the Pearson method, where green indicates strong correlation, yellow indicates weak correlation, and red indicates no correlation. E) Shows contribution and importance values from independent runs of correlated variable pairs (phase D) with MaxEnt, selecting the variable with the highest values, such as BIO14. F) Represents the final model with the 6 most explanatory variables for T. dimidiata, along with the results from MaxEnt: potential distribution map, AUC, and jackknife. (TIF) [file pntd.0013199.s001.tif]

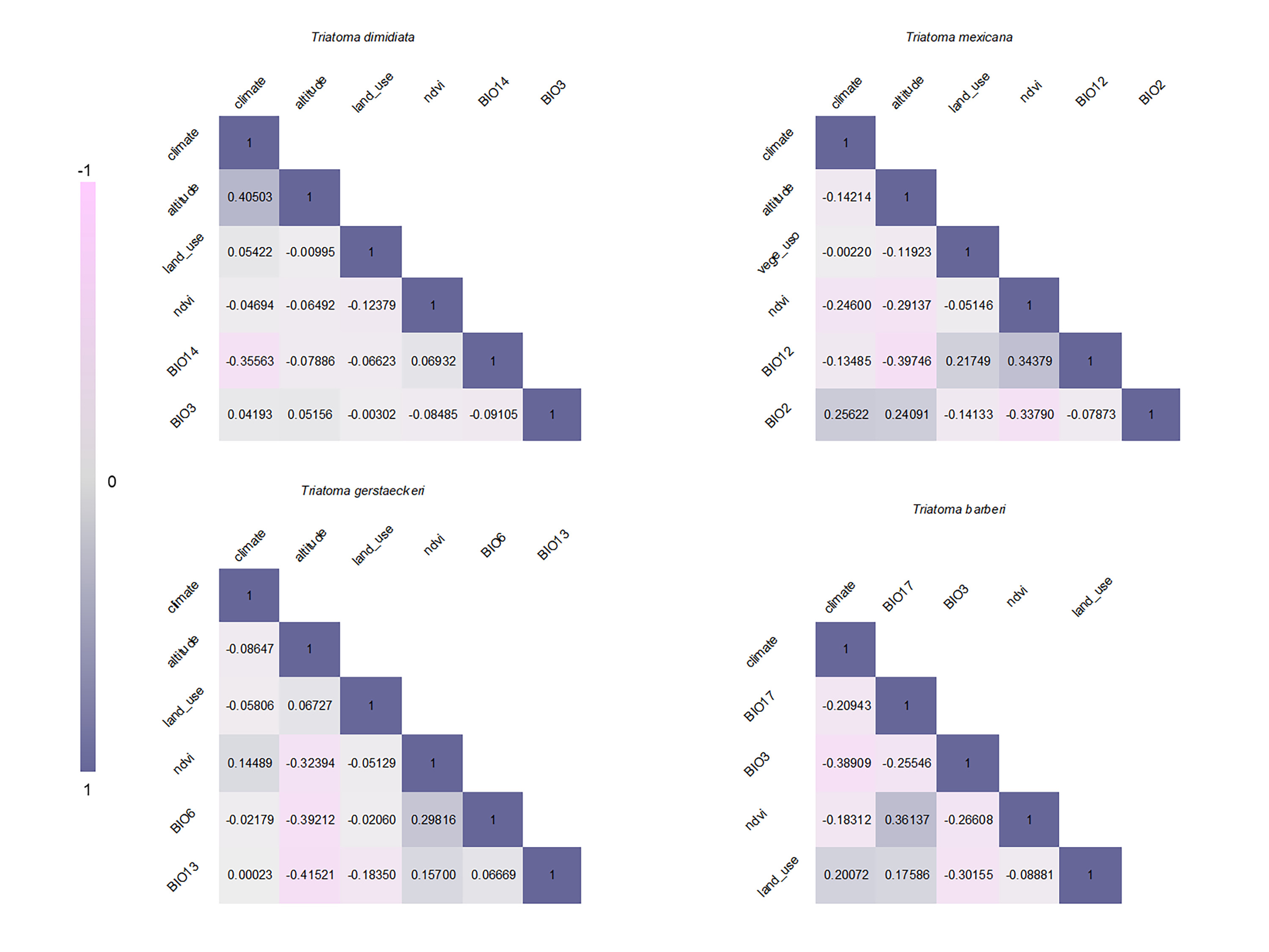

Supplement: S2 Fig — Each plot shows the Pearson correlation analysis of the final variables for each species, with correlation values in the cells. The color gradient indicates correlation strength: gray for no correlation (near zero), blue purple for strong positive correlation (one), and neon pink for strong negative correlation (-1). (TIF) [file pntd.0013199.s002.tif]

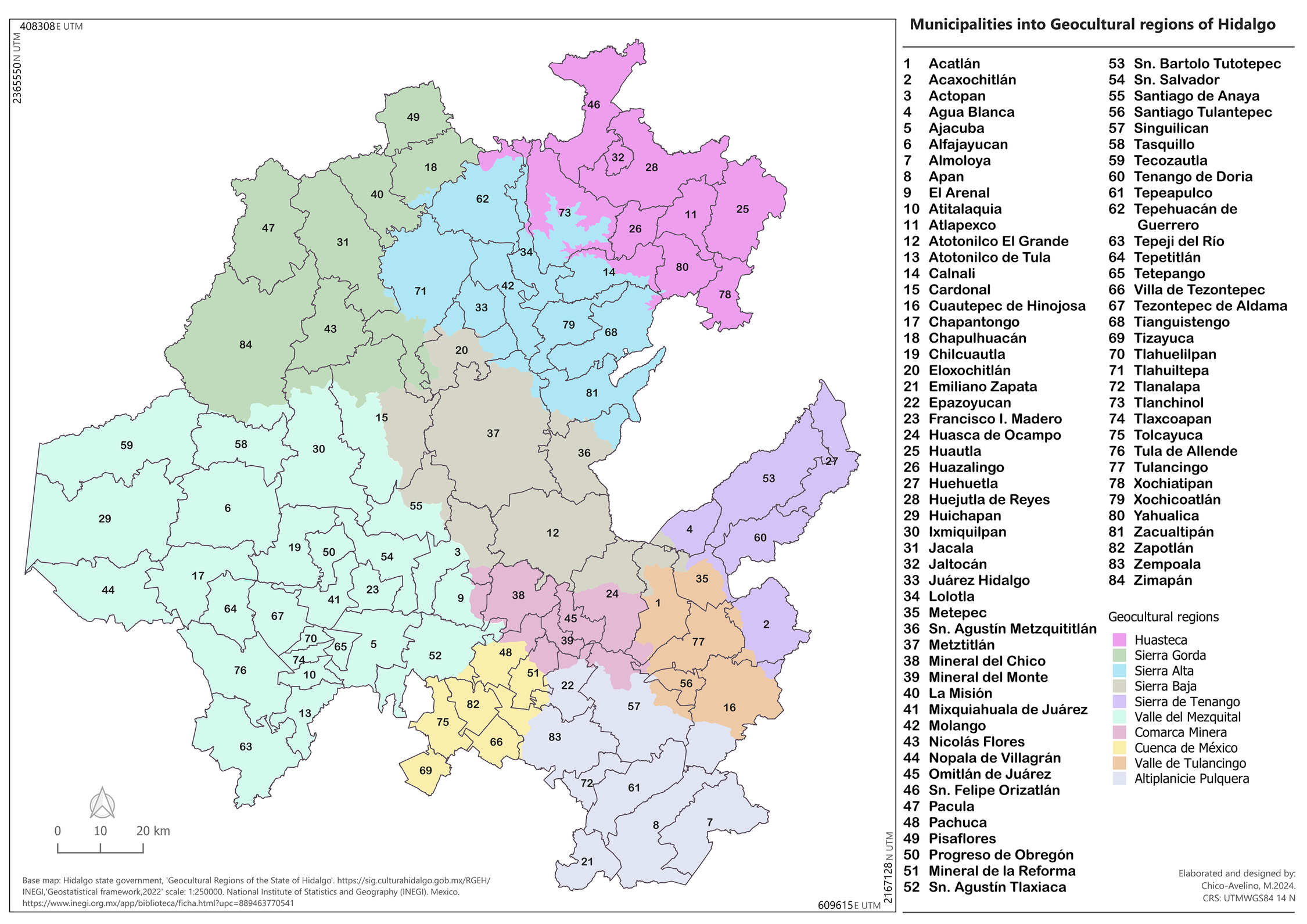

Supplement: S3 Fig — Municipalities are represented with a black border and an identifier number and are listed alphabetically on the right side of the map. The areas that comprise the geocultural regions that are represented in colors; in light fiusha color the Huasteca region, in light olive green the Sierra Gorda, in light blue the Sierra Alta, in sand color the Sierra Baja, in lilac color the Sierra de Tenango, in aqua green the Valle del Mezquital, light pink the Comarca Minera, the Cuenca de México in yellow, the Valle de Tulancingo in light orange and the Altiplanicie pulquera in light gray. Base map: Hidalgo State Government, Geocultural Regions of the State of Hidalgo (open access). [https://sig.culturahidalgo.gob.mx/RGEH/] | INEGI,‘Geostatistical framework, 2022’ scale: 1:250000. National Institute of Statistics and Geography (INEGI). Mexico. [https://www.inegi.org.mx/app/biblioteca/ficha.html?upc=889463770541]. This information is also included at the bottom of the map. (TIF) [file pntd.0013199.s003.tif]

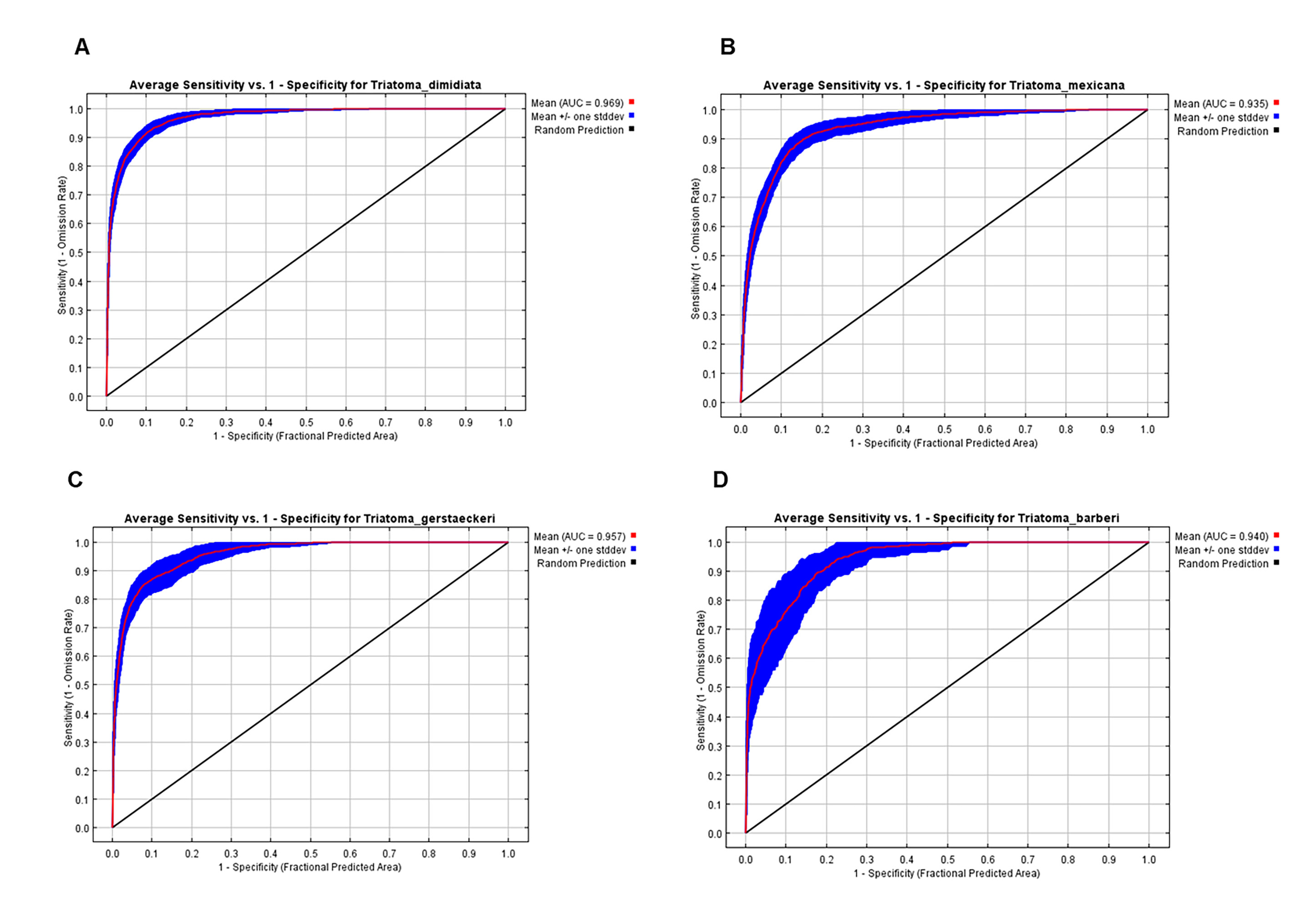

Supplement: S4 Fig — The red line represents the trend of the mean of the AUC values obtained in the 50 model replicates. The navy-blue area is the mean + /- one standard deviation of the AUC values obtained in the 50 model replicates. The black line corresponds to a random model that would have an AUC value of 0.5. (TIF) [file pntd.0013199.s004.tif]
